# Costs of reproduction in flowering plants

**DOI:** 10.1111/nph.70166

**Published:** 2025-05-07

**Authors:** Marcel E. Dorken, Mark van Kleunen, Marc Stift

**Affiliations:** ^1^ Department of Biology Trent University Peterborough ON K9L 0G2 Canada; ^2^ Department of Biology University of Konstanz Konstanz 78464 Germany; ^3^ Zhejiang Provincial Key Laboratory of Plant Evolutionary Ecology and Conservation Taizhou University Taizhou 318000 China; ^4^ Zhejiang Key Laboratory for Restoration of Damaged Coastal Ecosystems, School of Life Sciences Taizhou University Taizhou Zhejiang 318000 China; ^5^ Fraunhofer Institute for Molecular Biology and Applied Ecology IME Aachen 52074 Germany

**Keywords:** cost function, demographic costs of reproduction, life‐history trade‐offs, reproductive costs, sex‐allocation theory, somatic costs of reproduction

## Abstract

Costs of reproduction arise when investments into current reproduction reduce future reproductive fitness. Studies on reproductive costs use diverse approaches, including the analysis of gene expression, physiology, trade‐offs between reproduction and growth/survival, and the impact of reproductive investments on population growth. These studies demonstrate that reproductive trade‐offs have far‐reaching effects on plants, affect their fitness, and are therefore important for shaping the evolution of life histories. However, not all studies have detected costs of reproduction, and *c*. 90% of these were conducted in natural populations, where controlling for variation in plant resource status is challenging. For dioecious plants, there is a common perception that fruit production should result in greater costs of reproduction for females than males, but divergent reproductive costs between the sexes are not supported by studies of reproductive trade‐offs in dioecious plants. Other aspects of reproductive costs remain poorly understood, including ecological costs of reproduction, the fitness effects of reproductive trade‐offs involving growth or physiological processes, and how the male sex role influences reproductive costs. Progress will be enabled by the use of measurements that allow for easier comparisons across studies and by more clearly distinguishing between the processes that contribute to current vs future reproductive fitness.

**Table:** 

	Contents	
	Summary	55
I.	Introduction	55
II.	Background and practical considerations	56
III.	Systematic review of the costs of reproduction	61
IV.	Concluding remarks	65
	Acknowledgements	67
	References	67

## I. Introduction

The allocation of resources to reproduction can reduce a plant's capacity to reproduce again in the future, yielding a cost of reproduction. There is substantial evidence that such costs exist for plants, and these costs yield insights into the evolutionary processes shaping patterns of growth and survival in natural populations. The centrality of reproductive costs to plants' life histories has spurred many studies spanning a broad range of empirical approaches and taxonomic groups. These studies convincingly demonstrate that reproductive costs are an important element of the ecology and evolution of plant populations (Obeso, 2002). However, in spite of, or perhaps because of, the attention the topic has received, usage of the term ‘costs of reproduction’ has become increasingly diffuse since it was coined in the 1960s (Williams, 1966), and ‘costs of reproduction’ is now often used to refer to any type of reproductive trade‐off. At the same time, there has been a small but increasing number of studies that clearly demonstrate that the expression of reproductive trade‐offs affects fitness in a manner closely aligned with the original definition of the costs of reproduction (e.g. Calvo & Horvitz, 1990; Horvitz *et al*., 2010; Miller *et al*., 2012). Our primary aim in writing this review was to focus attention on the original conception of the costs of reproduction, incorporating insights from sex‐allocation theory with the intention that this would increase awareness of an understudied aspect of reproductive costs – those that arise from allocations to the male sex role. Unifying sex‐allocation theory with the ideas that underpin the costs of reproduction also links the fitness gains (Charnov, 1979) to the fitness costs of reproduction (Sletvold & Ågren, 2015), and a detailed examination of the extent to which costs of reproduction can differ between females and males in dioecious plants (Liu *et al*., 2022; Midgley, 2022).

## II. Background and practical considerations

### 1. Costs of reproduction

Costs of reproduction are defined as the reduction in expected future reproductive fitness arising from investments into current reproduction (Williams, 1966). These costs arise because resources are finite and the allocation of resources to reproduction reduces the amount of resources available for allocation to growth and/or survival (Acerenza, 2016; Mauro & Ghalambor, 2020), reducing the potential for future reproduction. Whether they are empirically detectable or not, costs of reproduction must exist: in the absence of reproductive costs, plants could allocate all of their resources to current reproduction without affecting their ability to grow and survive. This logic – that there must be an upper limit to the proportion of an organism's resources that can be allocated to reproduction without reducing its expected lifetime fitness – is what underpins the original conception of the costs of reproduction (Box 1). If we imagine a population of reproductively mature plants with identical, nonzero investment in current reproduction, but one plant allocates more resources into current reproduction than the others, one can ask: what is the (fitness) cost of that extra allocation? The plant might experience an immediate increase to the number of offspring it contributes to the next generation, but – because of trade‐offs between current and future reproduction – the expenditure of additional resources into current reproduction is justified only if it yields a net benefit to its lifetime reproductive fitness (Williams, 1966).

Box 1 **Costs of reproduction in hermaphrodites**

Costs of reproduction arise from the allocation of resources to reproduction that causes a reduction in expected future reproductive fitness via trade‐offs with survival and growth (Williams, 1966; Obeso, 2002). Williams (1966) defined costs in terms of changes to an individual's reproductive value that occur during a reproductive bout. Reproductive value is defined as the expected reproductive success of individuals of a given age and sex and denoted as Φ. The investment of resources into current reproduction reduces an individual's total reproductive value by an amount φ, yielding a residual reproductive value (RRV; Fisher, 1930; Pianka & Parker, 1975) of Φ−φ. That is, φ is the portion of Φ that is at stake in the current reproductive bout. An individual that allocates additional resources to flower or fruit production in a reproductive bout increases its value of φ by a factor *a*, at a cost to lifetime Φ by a factor *c*, yielding a new lifetime reproductive fitness, $\Phi^{\prime }$ (Williams, 1966):

$$\Phi^{\prime }=\left(1+a\right)\varphi +\left(1-c\right)\left(\Phi -\varphi \right)$$

Increased allocations to reproduction yield greater fitness than the default reproductive allocation whenever the cost, *c*, of increased reproductive allocation is smaller than the corresponding advantage, scaled by the plant's RRV: $c<a\cdot \varphi /\left(\Phi -\varphi \right)$. Note that the expression above is a simplified presentation of Williams' (1966) formulation intended to show the connection between reproductive costs and fitness. For example, we have ignored opportunity costs that can arise from the failure to make reproductive investments during the bout of reproduction associated with φ.

Even in the absence of resource‐based trade‐offs, there might often be limits to the extent to which extra reproductive expenditures enhance lifetime reproductive success. For a plant that already has a large investment in current reproduction, the production of additional pollen, flowers, fruits, etc. might have limited positive effects on the number of offspring it contributes to the next generation (Box 2). For example, the transfer of pollen among flowers within the same plant can reduce reproductive success via increased selfing and reduced pollen export (Lloyd, 1992; Karron & Mitchell, 2012), limiting the reproductive success of plants with large floral displays (Harder & Barrett, 1995). Under these conditions, each additional flower might contribute a diminishing amount to the value of current reproduction.

Box 2 **Realized costs of reproduction**

Realized costs of reproduction depend on the ecological properties of organisms and the environments in which they occur. These properties include average lifespans, the ability of organisms to recover reproductive resources, and the expected fitness pay‐offs from a given investment of resources into reproduction.

**Age‐specific costs of reproduction**

Residual reproductive values are based on age‐specific rates of mortality and reproduction. As a result, realized costs of reproduction depend on the age of reproduction and the intrinsic rate of increase in a phenotype (or genotype; Fisher, 1930). All else being equal, early reproduction justifies greater costs because it also has a greater effect on fitness than late reproduction. However, all is usually not equal. If fecundity and/or survival increase with age – particularly if size and age covary, as we generally expect for plants – then the allocation of resources to reproduction might instead increase with age (Charlesworth & Leon, 1976).

**Cost recovery**

A full accounting of reproductive investment includes potential resource gains from reproductive structures (e.g. via photosynthesis by floral or fruit tissues; Brazel & Ó'Maoiléidigh, 2019). Depending on how reproductive costs are measured, photosynthetic contributions or the partial recovery of resources from reproductive tissues might lead to inaccurate estimates of costs (Ashman, 1994). However, this does not mean that studies must examine resource gains from reproductive structures to estimate the costs of reproduction. What matters is the degree to which current reproduction affects future reproduction.

**Cyclical reproduction**

**Fig. B1:**
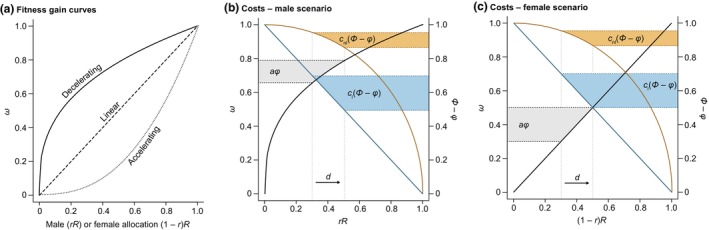
(a) Association between reproductive allocations and fertility can have various shapes. (b, c) Costs of reproduction depend on the association between the allocation of resources to reproduction and the effect of that allocation on expected future reproductive fitness (i.e. how allocations affect residual reproductive values). Because the two sex roles are thought to be characterized by different gain curves, we expect a different cost–benefit association between the sexes. The net benefit depends on the strengths of the trade‐offs arising from reproductive allocations and can be linear (blue), nonlinear concave‐down (orange), or nonlinear convex‐down (not shown; Sletvold & Ågren, 2015). If allocations to the female and male sex roles arise from a common resource pool, the shapes of the trade‐off functions are expected to be similar for the two sex roles. However, if the resources used for the female sex role differ from those used for the male role, the cost curves might also differ between the sex roles, increasing the scope for divergence in reproductive allocations between the sex roles. Note the difference between reproductive allocations and costs, at least for hermaphroditic plants. For example, if allocations to the male sex role are associated with a linear cost function, only small allocations to reproduction are justified (under the portion of the male gain curve with a steeper slope than that depicted for the linear trade‐off function). By contrast, if allocations to the female sex role are associated with a nonlinear cost function, a broad range of allocations yield greater fitness benefits than costs.

The costs and benefits of reproduction may not be constant from year to year and might, for example, depend on the abundance of seed‐eaters (Kelly & Sork, 2002). More generally, if reproductive fitness is a nonlinear (accelerating) function of reproductive allocations (Fig. B1), maximizing the fitness returns from reproduction might require reproductive investments that are not achievable every year. For example, if pollination efficiency increases with synchronized and above‐average reproductive effort among plants (including via the production of flowers and pollen), cycles of high and low reproductive effort (masting) might be evolutionarily favoured (Kelly & Sork, 2002). In terms of the costs of reproduction, masting yields interannual variation in the expression of costs and – particularly when masting cycles are synchronized among plants – frequency dependence of the benefits of current vs future reproduction.

**Other nonlinear effects**

The costs of reproduction scale with the fitness benefits from current reproduction, but these benefits can be a complex function of a plant's own reproductive investment. The previous example of masting may be a special case of this, but more generally, we expect that the fitness benefits of reproduction are a nonlinear function of the allocation of resources to reproduction (Charlesworth & Charlesworth, 1981). For example, if seed dispersal is spatially restricted, seedlings from the same plant will tend to compete with each other during establishment, reducing the net fitness benefits of reproduction to the maternal parent of those seeds (a form of local resource competition; Campbell, 2000). Similarly, if pollinators move pollen between neighbouring plants, pollen grains from the same plant will tend to compete with each other during the postpollination processes leading up to fertilization (a form of local mate competition; Lloyd, 1985). In both cases, this will result in diminishing fitness benefits as resource investment into reproduction increases. For example, plants that produce a larger‐than average amount of pollen or seeds would have lower per‐pollen or per‐seed fitness gains from that investment under local mate (or resource) competition. While it is difficult to account for these effects (Campbell, 2000), they are important for informing realized costs of reproduction.

### 2. Fitness in plants

Fitness is determined by the ‘objective fact of representation in future generations’ (Fisher, 1930). Accordingly, plant fitness is typically defined in terms of a phenotype's (or genotype's) propensity to transmit genes to future generations (Charnov *et al*., 1976; Lloyd, 1988; Charlesworth & Morgan, 1991). Most plants are hermaphroditic, and so fitness is determined by the joint contributions of the female and male sex roles. Fitness via the female sex role is determined by the production of ovules and the dispersal of seeds, while male fitness is determined by the production and dispersal of pollen (Lloyd, 1979). These reproductive roles involve the allocation of resources to primary reproductive characters (i.e. the gynoecium and the androecium) and to secondary reproductive characters that promote mating and dispersal (e.g. nectar and petals; Charlesworth & Morgan, 1991).

Resource allocations to primary and secondary reproductive tissues are expected to directly affect opportunities for reproduction via the two sex roles and therefore reproductive fitness (Box 3). This association between fitness and resource allocation could vary among plants and depend on aspects of their reproductive biology, including their ability to disperse seeds and pollen. A useful shorthand for describing this kind of variation is to characterize fitness as a mathematical function of reproductive allocations. These functions are referred to as fitness gain curves, and these curves are generally expected to differ between the female and male sex roles (Charnov, 1979; Klinkhamer *et al*., 1997). The gain‐curve approach has demonstrated that although population average fitness via the two sex roles is constrained to be equal, the optimal allocations to each sex role are not similarly constrained (Charnov, 1979; Charlesworth & Charlesworth, 1981; Crowley, 2008). For example, if the male gain curve decelerates more strongly than the female gain curve (Box 3), plants should, on average, allocate more resources to the female sex role (Sakai, 2000). That is, plants are expected to bias their reproductive allocations to the sex role that yields the largest benefits for a given reproductive investment (Charnov, 1979).

Unequal reproductive allocations between the sex roles can have important consequences for the expression of reproductive costs, at least for hermaphroditic plants. Because costs of reproduction scale positively with fitness gains from reproductive allocations, if one sex role is subject to more strongly decelerating fitness gains, then this limits the scope for the expression of reproductive costs for that sex role (Sakai, 2000). An extreme example of this is represented by highly selfing plants for which the male sex role is subject to strongly decelerating fitness gains – because there is no competition among plants for outcrossing opportunities, a plant should only produce enough pollen to fertilize its own ovules (Lloyd, 1985). For these plants, therefore, we expect strongly female‐biased sex allocations and greater costs of reproduction via the female sex role (Sakai, 2000).

These arguments regarding the potential for unequal costs and benefits of reproduction via the female and male sex roles appear to apply only to hermaphroditic plants. Costs of reproduction are expressed as reductions in expected future reproductive fitness, and the conditions under which the expected future fitness of females and males can diverge are thought to be limited (Williams, 1975) – or at least more limited than the degree to which costs of reproduction can diverge between the female and male sex roles of hermaphrodites (Crowley, 2008; and see Midgley, 2022). As we described below, reproductive allocations have been more intensively studied in dioecious plants than in other plants. One reason for this is that reproductive allocations and their effects on fitness are expected to be key factors in the evolution of dioecy. As Darwin (1877) himself suggested, and has since been confirmed by theory (Lloyd, 1976; Charlesworth, 1999) and experimentation (Dorken & Mitchard, 2008), the specialized allocation of resources to one sex role can help compensate for the loss of reproductive fitness via the other sex role (e.g. for the loss of the male sex role in female plants). The study of dioecious plants can also provide insights into the expression of reproductive costs via the female vs male sex roles (Obeso, 2002). However, as described below, care must still be taken to ensure that estimates of reproductive costs have similar effects on fitness in both sexes.

Perennial plants have overlapping generations, and for these plants, fitness has sometimes been defined as the transmission of genes through time (as opposed to generations; Zhang & Wang, 1994). Clonal growth further complicates definitions of fitness by providing another route by which plants can survive over time and, depending on the mode of clonal propagation, enable dispersal over short distances within populations or over longer distances resulting in colonization events (Klimešová & De Bello, 2009; Herben *et al*., 2016; Coughlan *et al*., 2017). Studies of the costs of reproduction in plants have included clonal propagation both as direct (Lin *et al*., 2016) and as indirect (Van Drunen & Dorken, 2012) contributors to reproductive fitness. Consensus on how clonal propagation contributes to current vs future reproduction is lacking, and we return to this point at the end of our review.

Box 3 **Costs vs benefits of reproduction**

Fitness for hermaphroditic (and monoecious) plants is the sum of gene transmission to future generations via the joint contributions from female and male sex roles. Using the above notation, we have the following: $\Phi_{t}=\Phi_{f}+\Phi_{m}$. For dioecious plants, females have $\Phi_{t}=\Phi_{f}$ and males have $\Phi_{t}=\Phi_{m}$. Because all individuals have a mother and a father (i.e. they have an egg and a sperm parent), average fitness via the female and male sex roles is equal (${\overset{`}{\Phi }}_{f}={\overset{`}{\Phi }}_{m}$) in both hermaphroditic and dioecious populations.

Fitness gains are not necessarily linear functions of investment into female or male sex roles. These functions are usually formulated as power functions of resource investment, with a proportion *r* of the total budget for current reproduction *R* allocated to the male sex role, and the remainder 1 − *r* allocated to the female sex role (Charnov, 1982; Zhang & Wang, 1994; Charlesworth, 1999). Accordingly, fertility gains from current allocations to the male sex role are $\varphi_{m}=\alpha_{m}\cdot {\left(\mathrm{rR}\right)}^{\mu }$, and fertility gains from the female sex role are $\varphi_{f}=\alpha_{f}\cdot {\left[\left(1-r\right)R\right]}^{\sigma }$, where α is a sex‐specific scaling factor, and μ and σ are exponents that determine the shape of the fitness function for the male and female sex roles (Fig. B1a). For hermaphroditic plants, a steeper – more strongly linear – fitness gain curve is usually expected for the female sex role (σ=1; Charnov, 1979), a scenario that corresponds with resource limitation of fertility (Bateman, 1948; Charnov, 1979). By contrast, the male sex role is generally expected to be subject to stronger mate limitation than the female sex role (Lehtonen, 2022), yielding a decelerating association between fertility and resource allocation (μ<1; Charnov, 1979). Although mate limitation or other nonlinear effects on fertility might also apply to the female sex role, it is thought that these effects are stronger for the male sex role, resulting in more strongly diminishing returns than for the female sex role of hermaphroditic plants (i.e. μ<σ; Zhang, 2006).

The costs of reproduction are expected to depend on reproductive effort – the proportion of resources allocated to reproduction. This dependence might take different forms for different plants (Calvo & Horvitz, 1990). Strong reproduction–growth trade‐offs could yield a linear trade‐off function (defined as $c_{l}$ and depicted using blue lines in Fig. B1b,c). For example, fruit production and total plant size appear to be subject to a 1 : 1 trade‐off in *Sagittaria latifolia* (Van Drunen & Dorken, 2012). Alternatively, allocations of resources to reproduction – particularly in long‐lived plants – might have minimal effects on future growth or survival (Sherman *et al*., 2019), yielding a concave association between resource allocation and the costs of reproduction (defined as $c_{\mathrm{nl}}$, depicted using orange curves in Fig. B1b,c; Reekie *et al*., 2002), and expressed in units of fitness (in units of Φ).

Costs of reproduction are justified if the increase in fitness from current reproduction (aφ) is greater than the costs. For a given reproductive allocation, the corresponding benefit aφ is greater when the gain curve is linear than when the curve decelerates, justifying greater costs of reproduction for the sex role with the steeper gain curve. If the shapes of the fitness gain curves for the female and male sex roles of hermaphroditic plants differ as expected, then so too could the costs. Fig. B1(b) outlines a possible scenario for reproductive benefits and costs for the male sex role assuming it is subject to decelerating fitness gains. In this scenario, $c_{l}\left(\Phi -\varphi \right)>\mathrm{a\varphi}>c_{\mathrm{nl}}\left(\Phi -\varphi \right)$, and an increase in allocation to male function by an amount *d* (from rR to ${\mathrm{rR}}^{\prime }$) is justified only if the fitness costs of reproduction are an even more strongly decreasing function of investment than the fitness gains (i.e. if the trade‐off function is as depicted by the orange curve in Fig. B1b). If there are stronger (linear) trade‐offs with reproductive investment, then the increased costs are not justified, and selection favours plants with reproductive allocations smaller than ${\mathrm{rR}}^{\prime }$. For the female sex role, a linear fitness gain curve is more likely to favour increased allocations to reproduction. For the scenario depicted in Fig. B1(c), females have broader scope for increased allocations to reproduction than males, with $c_{l}\left(\Phi -\varphi \right)=\mathrm{a\varphi}>c_{\mathrm{nl}}\left(\Phi -\varphi \right)$. However, note that this does not necessarily mean that all of *d* will be allocated to the female sex role – it is the product of fitness via both sex roles that is maximized (Charnov, 1979). Also note that the trade‐off functions depicted in Fig. B1(b,c) assume that all resources are available for allocation to current reproduction, and we expect shallower slopes for the trade‐off functions if only a small portion of total plant resources are used for reproduction.

These figures are intended to illustrate the difference between reproductive allocations and reproductive costs. That is, reproductive effort is not itself an estimate of reproductive costs. Costs are determined by the magnitude of the trade‐offs between reproductive allocations and growth or survival. As a result, even if the sex roles differ in their allocations, that does not necessarily mean that they differ in their costs, particularly if the two sex roles are subject to different types of trade‐offs. Indeed, the two sex roles might frequently be subject to different trade‐offs – if, for example, different types of resources limit the fertility of the two sex roles. For example, pollen production by the male sex role might often entail greater allocations of nitrogen than the female sex role, which might often entail greater allocations of biomass (Harris & Pannell, 2008; Van Drunen & Dorken, 2012).

As pointed out in the main text, there is less scope for divergent costs of reproduction for the female vs male sex roles in dioecious plants than in hermaphrodites. Fitness gain and trade‐off functions are subject to greater constraints in dioecious plants than for hermaphrodites. Dioecious plants are by definition fully outcrossing and subject to (at least) linear fitness gain curves (Seger & Eckhart, 1996). This, combined with equal average fitness for females and males, could constrain the degree to which the gain (and cost) curves can differ between the sexes.

### 3. Estimating costs of reproduction

A broad range of methodologies has been developed to examine reproductive trade‐offs (Obeso, 2002). These methods vary in the extent to which they capture trade‐offs between current and future reproductive fitness and include estimates of trade‐offs between reproduction and growth, including measures of physiological processes (Lambrecht‐McDowell & Radosevich, 2005), nutrient status (Harris & Pannell, 2008), plant size (Van Drunen & Dorken, 2012), and clonal propagation (Thompson & Eckert, 2004), postreproductive survival (Aragón *et al*., 2009), or future reproduction (Ågren & Willson, 1994). The most direct tests of the costs of reproduction have examined costs by estimating the finite rate of increase, λ (Calvo & Horvitz, 1990; Horvitz *et al*., 2010), a close proxy for fitness (Charlesworth, 1994; Caswell, 2006). Costs have also been assessed at various levels of plant modularity, including at the level of branches (or smaller collections of modules; e.g. Kawamura & Takeda, 2006; Ueno *et al*., 2006), ramets (the individual shoots that make up the genet, or genetic individual, of clonal plants; Ida *et al*., 2015), and whole plants, including genets (Shefferson *et al*., 2003), shrubs (Aragón *et al*., 2009), and trees (Barringer *et al*., 2013).

In general, studies of the costs of reproduction involve examinations of one or both of two major classes of reproductive trade‐offs: studies of (1) trade‐offs between reproduction and plant growth (or the physiological processes supporting plant growth); or (2) trade‐offs more closely aligned to plant fitness, such as trade‐offs between reproduction and survival or future reproduction (Obeso, 2002). Accordingly, we follow previous reviews of the costs of reproduction in plants in distinguishing between two types of estimates of reproductive costs: estimates of trade‐offs between reproduction and somatic growth (somatic costs of reproduction; Tuomi *et al*., 1983), and trade‐offs between reproduction and expected future fitness (i.e. trade‐offs between reproduction and survival or future reproductive success, typically referred to as demographic costs of reproduction; Horvitz & Schemske, 1988; Obeso, 2002). Studies of these two different types of trade‐offs, which we will refer to using the short‐hands ‘somatic’ and ‘demographic’ costs of reproduction, vary in the directness with which they provide insights into the fitness effects of reproductive allocations, and therefore into the cost of reproduction as they were originally defined as a trade‐off between current and future reproductive fitness (Box 4). Studies of somatic costs of reproduction provide insights into the effects of reproductive allocations on growth, and thereby into the mechanisms by which reproductive allocations might filter through plant life histories to affect fitness (e.g. McDowell & Turner, 2002). Studies of the demographic costs of reproduction, by contrast, provide direct insights into the fitness costs of reproductive investments, but may provide limited insights into the specific processes occurring within plants by which reproductive allocations affect future fitness (Calvo & Horvitz, 1990).

Box 4 **Somatic vs demographic costs of reproduction**

**Fig. B2:**
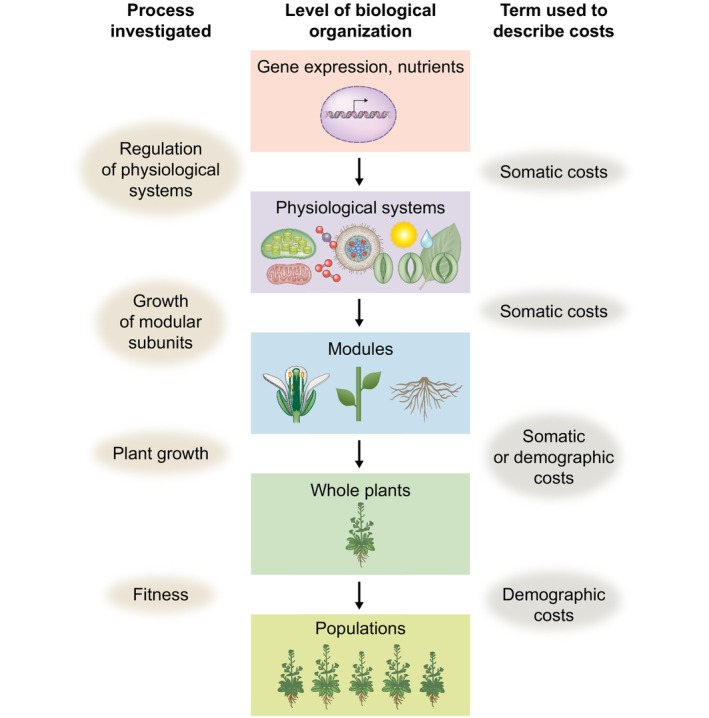
Costs of reproduction have been assessed across hierarchical levels of biological organization. Studies of processes associated with lower levels of biological organization are typically associated with estimates of the somatic costs of reproduction. By contrast, processes associated with higher levels of biological organization are typically associated with estimates of the demographic costs of reproduction.

The terms used to describe the costs of reproduction vary in the degree to which they address the core idea of the costs of reproduction – the reduction in future reproductive fitness caused by the allocation of resources to current reproduction (Fig. B2). Studies involving measurements made at lower levels of biological organization provide insights into the mechanisms underlying reproductive costs, while studies involving measurements made at higher levels more closely correspond to tests of the idea that reproductive trade‐offs affect plant fitness. Studies of reproductive trade‐offs at the whole‐plant level include estimates of somatic or demographic costs of reproduction. Studies conducted at the population level over multiple years have examined how reproduction allocations contribute to the finite rate of population increase, *λ*.

Studies of the costs of reproduction that involve analyses of variation in patterns of gene expression or genetic variation among individuals can also be characterized using the somatic/demographic dichotomy. A small number of studies of patterns of gene expression in plants with different reproductive phenotypes have provided insights into the processes regulating trade‐offs between reproduction and growth (Zemp *et al*., 2016; Cossard *et al*., 2019), and therefore into the processes regulating somatic costs of reproduction. Alternatively, genetic marker studies have provided insights into the demographic costs of reproduction by revealing negative pleiotropic effects of loci associated with reproductive allocations on fitness‐related traits, such as survival (Anderson *et al*., 2014; and see Remington *et al*., 2013).

## III. Systematic review of the costs of reproduction

### 1. Scope of the systematic review

Previous reviews of the costs of reproduction in plants have reported widespread reproductive trade‐offs, affecting plant growth, survival, and future reproduction (Charlesworth & Morgan, 1991; Obeso, 2002). Reproductive trade‐offs have been identified across experimental and observational studies and across a wide range of plant growth forms (Obeso, 2002). However, not all studies have detected reproductive costs. Accordingly, one of our objectives was to identify whether different types of studies, or the use of different types of metrics, might affect the identification of reproductive costs. In his review, Obeso (2002) identified several knowledge gaps in the understanding of the costs of reproduction and made a series of recommendations for future research, including the need for direct estimates of fitness to study reproductive costs and for tests of the size dependency of reproductive costs. Our second objective was to focus part of our review on studies that used estimates of fitness to examine reproductive costs and that tested for size‐dependent costs of reproduction. Finally, because there is a theoretical expectation for similar costs of reproduction between females and males in dioecious plants, we also reassessed the commonly reported finding that costs of reproduction are greater for females than for males.

### 2. Inclusion criteria

Systematic reviews, which have clear criteria for inclusion of studies, have become a standard way to review literature in ecology, as they reduce biases and increase replicability (Hillebrand & Gurevitch, 2016). We followed the Preferred Reporting Items for Systematic reviews and Meta‐Analyses (PRISMA) guidelines for conducting systematic reviews (Page *et al*., 2021), focusing our attention on the recent literature, particularly those studies that had not been considered in previous reviews of the costs of reproduction (Charlesworth & Morgan, 1991; Obeso, 2002). We provide detailed methods in Supporting Information Methods S1, but briefly, we searched the Web of Science Core Collection on 13 February 2023 using the following search phrase: ALL = (“costs of reproduction” OR “cost of reproduction” OR “reproductive cost” OR “reproductive costs”) AND ALL = (plant OR tree OR shrub OR perennial OR dioec* or monoec* OR flower OR pollen OR ovule) AND PY = (2002–2023). We also added any articles that cited Obeso (2002) that did not appear in our first search using the Web of Science Cited Reference Search tool. Because a more complete analysis was needed to address our aim of re‐examining the evidence for differences in the costs of reproduction between females and males, we included all papers listed in Obeso's (2002) appendix 2 (all dioecious and other gender‐dimorphic plants considered in that review) in the list of records screened for inclusion. This search yielded 1219 unique records.

To mitigate bias associated with the screening of articles, we used software that deployed a machine‐learning algorithm to sort the records according to their fit with the inclusion criteria (van de Schoot *et al*., 2021). The algorithm was initially trained using a set of 10 suitable (according to our inclusion criteria) and 10 unsuitable records from the list of records obtained. The program presents articles to be screened showing only the article title and abstract in order of their fit, which is updated as articles are screened. During screening, we excluded studies that did not explicitly examine trade‐offs involving reproduction. After screening, a total of 178 studies representing 177 different species were included in our review. The complete list of studies and the R scripts used to calculate the summary statistics presented below are available at doi: 10.6084/m9.figshare.26388499.

The detectability and/or magnitude of reproductive costs are expected to vary across studies for a number of reasons, including the types of plants studied and the ways in which costs are evaluated. First, the magnitude of reproductive costs is expected to vary across plant life histories, with annuals having maximal costs and long‐lived plants having higher residual reproductive values and therefore lower costs per reproductive bout (Silvertown & Charlesworth, 2001). Second, studies in natural populations involve plants growing under environmentally heterogeneous conditions that may differ in age, size, or resource status. Under these conditions, identifying costs might be challenging (Reznick, 1985), not least because variation in resource status among plants can mask the expression of trade‐offs (van Noordwijk & de Jong, 1986; Venable, 1992). Third, trade‐offs might be obscured by resource sharing among modules. As a result, trade‐offs examined at the modular or ramet level might not reflect those affecting entire plants (van Kleunen *et al*., 2000; Bañuelos & Obeso, 2004). Fourth, although observational studies frequently detect reproductive trade‐offs, the controlled manipulation of variables and reduction in confounding factors involved in experimental studies should provide more robust insights (Obeso, 2002). Finally, multiyear studies might be necessary to detect reproductive costs, particularly if plants use stored resources for reproduction instead of recently acquired resources, such as photosynthates in leaves (Ida *et al*., 2012). We tested whether these five factors influenced the detectability of reproductive costs using logistic regression. Because plant longevity is often not known or not reported in the study, we used woodiness as an indicator of plant longevity (Chondol *et al*., 2023). Logistic regression parameters and their significance were estimated using the *glm* function in R (v.4.4.2.; R Core Team, 2024).

### 3. Overview of studies

Approximately half of the 178 studies included in this review (81 studies, 46%) measured some aspect of the demographic costs of reproduction by investigating trade‐offs between current and future reproduction and/or survival. However, many more studies (150, 84%) involved measures of the somatic costs of reproduction. Most studies of the costs of reproduction involved the examination of plants in natural populations, with 133 (75%) studies in natural populations, of which 70 (39%) included estimates of the demographic costs of reproduction. By contrast, studies conducted in controlled environments (e.g. common gardens or glasshouses) tended to involve estimates of the somatic costs of reproduction, with 31 studies of somatic costs among 46 studies conducted in controlled environments. Most studies conducted in natural populations involved the analysis of phenotypic correlations (95 of 133 studies, or 71%). Many fewer studies involved experimentally manipulated plants in either natural populations (53 studies, 30%) or controlled environments (32 studies, 18%). Of the 85 experimental studies, most (58 studies) involved a manipulation of reproductive investments (e.g. flower bud removal). Most experimental studies were conducted using herbaceous plants (60 studies, 71%). The frequent use of herbaceous plants in experimental studies appears to have enabled analysis of the fitness costs of reproduction. Of these 60 experimental studies of herbaceous plants, almost two‐thirds (35 studies, 58%) included estimates of the demographic costs of reproduction. A total of 177 plants examined across studies were gender dimorphic (61, 34%). By comparison, dioecy and other gender dimorphisms are relatively rare – 5–6% of flowering plants are dioecious, and there are even lower frequencies of gynodioecy and androdioecy (Renner, 2014).

**Fig. 1:**
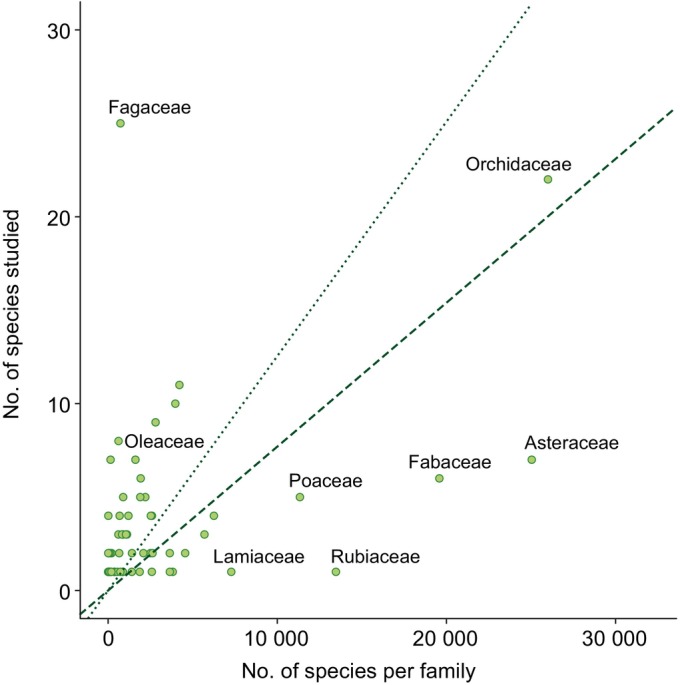
Costs of reproduction are understudied in the most diverse plant families. The dotted line indicates the null expectation if the number of species studied for reproductive costs per family was proportional to the number of species per family. The dashed line indicates the null expectation if the number of species studied was proportional to the number of angiosperm species.

A total of 65 families were represented among the plants studied for costs of reproduction, but the largest families tended to be understudied in relation to the number of species in the family (Fig. 1; this result changes slightly when the number of studied species is compared against the total number of angiosperm species, here assumed to be *c*. 300 000, with orchids slightly over‐represented). Two relatively small plant families (Fagaceae and Betulaceae) were over‐represented. For the Fagaceae, this over‐representation was attributable to the repeated study of two woody genera (*Quercus*: *n* = 17; *Fagus*: *n* = 8).

### 4. Costs are less detectable in natural populations

**Fig. 2:**
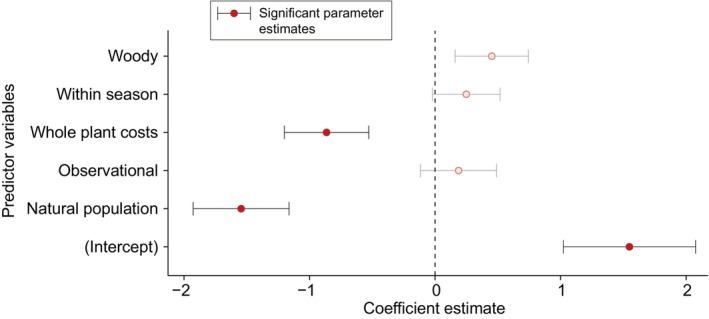
Results of a logistic regression used to examine whether the ability to detect costs of reproduction in plants varies across contexts and approaches. Studies of the costs of reproduction in natural populations, and studies of entire plants, more commonly reported that costs were not detected than did studies that were conducted in controlled environments or studies of smaller subunits within plants, for example branches. The frequency with which costs of reproduction were reported from observational (vs experimental) studies, woody (vs herbaceous) plants, or from studies of plants or within (vs between) flowering seasons, did not vary significantly. Significant parameter estimates are indicated by red (filled) symbols. Values are the logistic regression parameter estimates ± SE. Parameter estimates and other details of the logistic regression are reported in Supporting Information Table S1.

The majority of included studies reported results that were consistent with the expression of reproductive costs (148 of 178 studies, 83%). Among the studies that failed to detect costs of reproduction, 89% (25 of 28 studies) were conducted in natural populations (Fig. 2; Table S1). The greater difficulty in detecting reproductive costs in natural populations might reflect the challenges of studying trade‐offs in settings where variation in plant resource status, size, and age is not controlled before the start of the experiment. Indeed, it is now well established that heterogeneity in the environment and age structure can lead to positive correlations between traits subject to an underlying trade‐off, and so for which negative correlations are expected (van Noordwijk & de Jong, 1986; Roff & Fairbairn, 2007; Morrissey *et al*., 2010).

That costs of reproduction can be harder to detect in natural populations does not mean that one should not study costs in the wild. Indeed, the studies that most clearly demonstrate costs of reproduction – as a fitness‐based trade‐off between current and future reproduction – have been conducted in natural populations (e.g. Horvitz *et al*., 2010). Such studies have shown that costs of reproduction affect fitness – estimated as the finite rate of population growth, λ – via trade‐offs between reproduction and growth (Horvitz *et al*., 2010; Lin *et al*., 2016; Kellett & Shefferson, 2018). Studies conducted in natural populations have also provided insights into the expectation that costs of reproduction should be weaker in long‐lived plants than in plants with shorter lifespans (Charlesworth, 1994). Long‐term studies conducted in natural populations have been useful in testing this expectation. For example, in the long‐lived terrestrial orchid *Orchis purpurea*, which has an estimated lifespan of up to 60 years, costs of reproduction are expressed as trade‐offs between reproduction and plant size but not – at least not directly – survival (Miller *et al*., 2012). This reproduction–growth trade‐off represents a cost of reproduction because reproductive output is size‐dependent for this plant, yielding an indirect trade‐off between current and future reproduction (Miller *et al*., 2012). By contrast, for the short‐lived shrub *Helianthemum squamatum*, regeneration from a persistent soil seed bank appears to be the main driver of population growth. Accordingly, strong selection on seed fecundity is expected, and costs of reproduction are expressed as a trade‐off with survival, not future fecundity (Aragón *et al*., 2009).

Two other aspects of the logistic regression are noteworthy. First, whether costs were assessed at the whole‐plant level or not also affected the detection of reproductive costs (Fig. 2). In general, studies conducted at the whole‐plant level were less likely to detect costs than studies of trade‐offs occurring within modular subunits (e.g. shoots or branches). A small number of studies have examined reproductive costs at multiple hierarchical levels to evaluate the extent to which trade‐offs expressed at the branch level correspond with whole‐plant estimates of reproductive costs (Bañuelos & Obeso, 2004; Tozawa *et al*., 2009; Sánchez‐Humanes *et al*., 2011; Wang *et al*., 2015). There are too few studies to draw general conclusions about the correspondence in estimates of reproductive costs across hierarchical levels, but it is clear that trade‐offs expressed within smaller subunits do not necessarily scale up to reflect whole‐plant reproductive costs (Bañuelos & Obeso, 2004; Sánchez‐Humanes *et al*., 2011). Second, the model intercept was significantly different from zero, suggesting that the model reference case was associated with > 50% probability of detecting reproductive costs (where the reference case corresponds with herbaceous plants that were experimentally manipulated in controlled environments and with the study of reproductive costs between flowering seasons at levels below that of entire plants).

### 5. Size‐dependent costs of reproduction

Only 16 studies considered the size dependency of reproductive costs in some way. Size dependence of reproductive costs has been evaluated using a variety of techniques, including through the use of plant size in the analysis of vital rates for the estimation of λ (Calvo & Horvitz, 1990; Jacquemyn *et al*., 2010; Miller *et al*., 2012; Kellett & Shefferson, 2018), meristem‐level analyses of growth–reproduction trade‐offs among plants of different sizes (Miller *et al*., 2008), and direct tests of variation in reproductive costs for plants of different sizes (Sletvold & Ågren, 2011, 2015; Tye *et al*., 2020; Sun *et al*., 2022). They have also been evaluated for a range of plant types, including herbaceous plants (e.g. Miller *et al*., 2008; Jacquemyn *et al*., 2010), shrubs (e.g. Kawamura & Takeda, 2006), and trees (e.g. Oddou‐Muratorio *et al*., 2021). For studies conducted in natural populations, size and age were usually confounded. This range of approaches and the possibility that size and age were confounded in the evaluation of reproductive costs complicates comparisons across studies. Moreover, some of these studies either failed to detect costs of reproduction altogether (Shefferson *et al*., 2011), or detected costs of reproduction that were independent of plant size (e.g. Sletvold & Ågren, 2015).

Mixed findings regarding the size dependency of the costs of reproduction are not unexpected. On the one hand, size‐dependent allocations of resources into reproduction can mask the costs of reproduction if plants prioritize resource allocations to survival (Zhang & Jiang, 2002). Under this scenario, reproductive allocations can be independent of allocations to survival, making reproductive costs hard to detect (Zhang & Jiang, 2002). On the other hand, allocations to reproduction and survival might both vary with plant size, and under these conditions, there is scope for large plants, or plants with larger resource budgets, to have lower costs of reproduction than small plants (Klinkhamer *et al*., 1997). In general, therefore, variation in plant size might make costs of reproduction hard to detect or result in lower costs of reproduction for large than for small plants.

### 6. Fitness costs of reproduction

Detailed demographic studies of plant populations remain a relatively rare approach for the study of the costs of reproduction. The approach, by which reproductive costs are inferred via the effects of reproduction on the finite rate of population increase, λ, was first introduced in the 1990s (Calvo & Horvitz, 1990), with eight studies using this approach since then (García & Ehrlén, 2002; Horvitz *et al*., 2010; Jacquemyn *et al*., 2010; Shelton, 2010; Shefferson *et al*., 2011; Miller *et al*., 2012; Lin *et al*., 2016; Kellett & Shefferson, 2018). Although rarely used, this approach provides the most direct assessment of the costs of reproduction in plants. For example, for *Lathyrus vernus*, data from 24 years of observations and 2 years of experimentally increased reproductive effort via supplemental pollination revealed a negative association between current reproduction and future size (Horvitz *et al*., 2010). Population growth rates for this plant depend on the frequency of large, reproductive individuals. As a result, costs of reproduction in this plant are expressed as a trade‐off between reproduction and growth, with the (large) plants that reproduce less likely to remain large and reproductive in the subsequent flowering season. Demographic studies, such as those conducted on *L. vernus*, provide empirical demonstrations of a core principle of the costs of reproduction: costs of reproduction yield a net benefit to fitness when they are more than offset by enhanced offspring production. This core principle was also demonstrated by a 3‐year study of neotropical *Asclepias curassavica* (Kellett & Shefferson, 2018). For this long‐lived herbaceous plant, costs were expressed as reductions in survival and growth for reproductive compared with nonreproductive plants and the potential benefits of seed production per reproductive bout were measured by tracking temporal variation in the probability of seedling establishment. Detailed studies of the demographic costs of reproduction like these have provided insights into the ecological and evolutionary processes affecting sex ratios in dioecious plants (Shelton, 2010), the relative contributions of clonal propagation and sexual reproduction for population persistence (Lin *et al*., 2016), the evolution of delayed reproduction (Kellett & Shefferson, 2018), and the relative importance of herbivory to vegetative vs reproductive tissues for plant fitness (García & Ehrlén, 2002). These broad applications underscore the central role of reproductive costs in plant ecology and evolution.

### 7. Underlying mechanisms

Because costs of reproduction involve changes in organismal fitness, they are necessarily expressed in the future, after reproduction has occurred. As a result, reproductive costs arise via other, more proximate trade‐offs that occur at the time that reproductive allocations are made. The most immediate of these trade‐offs involve changes in patterns of gene expression, physiological processes, and the allocation of meristems to growth vs reproduction. Studies of these proximate mechanisms underlying reproductive trade‐offs have included measurements of nutrient concentrations (Reekie *et al*., 2002; Harris & Pannell, 2008; Van Drunen & Dorken, 2012; Han *et al*., 2014; Sánchez Vilas & Retuerto, 2016; Long *et al*., 2021), inferred rates of carbon acquisition or its movement within plants by estimating photosynthetic rates (Miyazaki *et al*., 2002; Verdú, 2004; Wheelwright & Logan, 2004; Horibata *et al*., 2007; Kudo & Ida, 2010; Shibata & Kudo, 2016) or by tracking carbon isotopes (Verdú, 2004; Ida *et al*., 2012; Sunmonu *et al*., 2013; Han *et al*., 2016). Studies using a combination of these approaches enable a comprehensive analysis of the proximate processes regulating reproductive costs. For example, an experimental manipulation of flower production for two species of *Rubus*, one of which is invasive where the study was conducted, showed that reproduction was associated with reduced photosynthetic rates (McDowell & Turner, 2002). Lower photosynthetic rates occurred primarily when nitrogen was being translocated to fruits, and as indicated by measures of carbon isotope ratios, reproductive plants experienced greater water stress than nonreproductive plants. Because these plants are highly clonal, differences in the magnitude of these trade‐offs between reproduction and growth help explain the spread of invasive *Rubus* (McDowell & Turner, 2002).

The proximate trade‐offs regulating reproductive costs can filter through plant life histories in a complex manner. For example, the measurement of reproductive costs can be obscured by the presence of storage organs and the amount of time elapsed between resource acquisition and the allocation of those resources to reproduction. For *Corydalis ambigua*, an herbaceous spring ephemeral, leaf photosynthates do not appear to be needed for fruit production, which depends on the translocation of previously acquired photosynthates held in storage organs (Kudo & Ida, 2010). For long‐lived trees, such as oaks, the appearance of trade‐offs between growth and reproduction is not necessarily indicative of costs of reproduction (Knops *et al*., 2007). Instead, the routes by which resources used for reproduction affect growth and subsequent reproduction in oaks are more complex – interactions between the environmental availability of resources, reproduction, and growth yield costs of reproduction that are only apparent across years, and when studied at the whole‐tree level (Barringer *et al*., 2013). Studies like these highlight the complexities that might need to be accounted for when studying costs of reproduction and that an integrated, whole‐plant approach might often be necessary to identify somatic costs.

The mechanisms underlying the expression of demographic costs of reproduction have been examined by studying how the loci influencing reproductive allocations impact survival and future reproduction. Specifically, if there are costs of reproduction, alleles associated with greater reproductive allocations are expected to also be associated with reduced survival or future reproduction (i.e. the loci affecting reproductive allocations are expected to exhibit antagonistic pleiotropy). A study of *Boechera stricta* that specifically aimed to identify pleiotropic interactions between growth and reproduction using a modification of the quantitative trait locus (QTL) mapping approach found that specific QTL alleles associated with greater reproduction also reduced subsequent survival (Anderson *et al*., 2014), consistent with the expectation that costs of reproduction are mediated by antagonistic pleiotropy. However, for closely related *Arabidopsis lyrata*, analysis of QTLs associated with reproduction revealed no negative pleiotropic effects with growth or survival. Instead, costs of reproduction were expressed as meristem‐based trade‐offs, with costs – estimated as negative phenotypic associations between reproduction and growth – resulting from the timing of rosette branching events (Remington *et al*., 2013). Meristem‐based trade‐offs have been inferred to be important in regulating the costs of reproduction for plants representing a range of growth forms, including rosette forming plants, such as *A. lyrata*, the branching cladode growth form of *Opuntia imbricata* (Miller *et al*., 2008), and large trees, such as *Nothofagus* spp. (Torres *et al*., 2016). Meristem proliferation, growth, and reproduction all rely on the availability of resources, and so even if costs of reproduction appear to be mediated by meristem limitation, underlying antagonistic associations between loci might still play important roles if these loci affect the deployment of resources to current reproduction vs survival and growth.

### 8. Costs in gender‐dimorphic plants

Studies of gender‐dimorphic plants provide no clear evidence for general differences in the costs of reproduction between the sexes (Table 1). Previous reviews have argued that sexual dimorphisms in growth, survival, and reproductive allocations are indicative of differences in the costs of reproduction between females and males. The argument is typically framed as follows: (1) females of dioecious plants have greater reproductive effort than males because they produce fruits and males do not; (2) greater reproductive effort by females translates to greater reproductive trade‐offs for females than males, leading to sex‐differential costs of reproduction; and (3) these differential costs of reproduction are expressed as differences in morphology, life history, and habitat preferences between females and males (i.e. as sexual dimorphisms). However, it has long been known that the fact that females produce fruits and males do not is not the cause of sexual dimorphisms. As pointed out by Lloyd & Webb (1977), ‘the mere fact that’ females invest significantly in seed and fruit maturation and fertilization does not necessarily mean they have greater costs of reproduction. If it were beneficial, natural selection would favour females that allocate fewer resources to sexual reproduction by producing fewer flowers than males (Delph *et al*., 2005). Indeed, the data summarized in Table 1 indicate that fruit production by females is not by itself a cause of general differences in reproductive trade‐offs between the sexes.

**Table 1:** Summary of results of studies of gender‐dimorphic plants.

Growth form	NS	M > F	F > M	Depends
Woody	8	2	10	0
Herbaceous	6	4	12	3
All plants	14	6	22	3

Among the relatively small number of studies that compared somatic and/or demographic costs of reproduction between the sexes through the examination of reproduction trade‐offs, there were roughly four times as many instances for which females were found to bear the greater cost (F > M) than for which males were found to have greater costs (M > F), with similar patterns for both woody and herbaceous plants. However, fewer total studies found greater costs of reproduction for females than found no significant differences in reproductive costs between the sexes (NS), that males had greater costs, or that the sex that bore the greater cost depended on the cost metric used (depends; e.g. whether costs were estimated using units of biomass or nitrogen content). F, female; M, male.

Studies of animals have clarified the importance of sexual selection in driving the evolution of sexual dimorphisms (Fairbairn *et al*., 2007; Cox & Calsbeek, 2009; and see Barrett & Hough, 2013). Sexual selection – nonrandom variance in mating success (Kokko & Jennions, 2008) – may be widespread in flowering plants (Moore & Pannell, 2011; Tonnabel *et al*., 2021) and could therefore be responsible for the evolution of sexual dimorphisms in plants, as it is in animals. In plants, sexual selection can operate on reproductive traits, such as flower size (Delph & Herlihy, 2012) and number (Dorken & Perry, 2017), but might also operate on nonreproductive traits, such as plant height (Gervasi & Schiestl, 2017). Sexual selection contrasts with other forms of selection that might take place during reproduction, including compatibility selection and fecundity selection. Fecundity selection, which arises from nonrandom variation in reproductive success, has also been associated with the evolution of sexual dimorphism (Fairbairn *et al*., 2007), but it appears to have substantially weaker effects on the evolution of sexual dimorphisms than sexual selection (Cox & Calsbeek, 2009). Moreover, sexual selection can be responsible for differences in reproductive costs between the sexes. For example, in animals strong sexual selection among males can result in substantial life‐history trade‐offs that reduce their survival, increasing the value of current reproduction and yielding higher costs of reproduction for males than for females (Lemaître *et al*., 2020).

Studies linking costs of reproduction, sexual dimorphism, and sexual selection are rare, but have been conducted for three plants. The clearest connection between divergent costs of reproduction to sexual dimorphisms and sexual selection comes from studies of *Silene latifolia*, for which costs of reproduction are greater for males, not females. Flower production is the most sexually dimorphic trait for *S. latifolia*, with males producing more flowers than females (Delph & Herlihy, 2012). Greater flower production by males is driven by strong sexual selection on mating success (Delph & Herlihy, 2012; Delph *et al*., 2022), at a cost to growth (Delph *et al*., 2005) and survival (Delph *et al*., 2022). However, studies of *Mercurialis annua* and *Sagittaria latifolia* provide a contrasting set of examples. Both plants are characterized by strong sexual selection on mating success for males (Tonnabel *et al*., 2019; Kwok & Dorken, 2022), substantial reproductive trade‐offs for males that involve greater allocations of nitrogen to reproduction than females, and sexual dimorphisms for secondary‐sex characters that affect pollen dispersal – elongated inflorescence stalks for males of wind‐pollinated *M. annua* (Eppley & Pannell, 2007) and larger floral displays for males of animal‐pollinated *Sa. latifolia* (Dorken & Perry, 2017). For both plants, however, the costs of reproduction do not appear to differ between females vs males – the magnitude of reproductive trade‐offs depends on the metric used to estimate costs (Harris & Pannell, 2008; Van Drunen & Dorken, 2012), the primary sex ratios are balanced (Dorken & Barrett, 2004; Russell & Pannell, 2015), and for clonal *Sa. latifolia*, genet sex ratios are balanced when averaged across populations (Yakimowski & Barrett, 2014). All else being equal, balanced sex ratios imply balanced reproductive opportunities for females vs males over time, and similar changes in the future reproductive value of females and males following reproduction (Midgley *et al*., 2019). That is, strong sexual dimorphisms in *M. annua* and *Sa. latifolia* do not appear to be driven by divergent costs of reproduction between the sexes in spite of strong sexual selection.

## IV. Concluding remarks

Our review highlights the existence of gaps between the conceptual understanding of reproductive costs and empirical testing. One of these gaps arises from the fact that although, on average, half of plant fitness comes from the male sex role, very little is known about costs arising from the male sex role. A second gap involves the lack of data addressing the shape of cost functions in plants (Reekie *et al*., 2002; Sletvold & Ågren, 2015). Studies of the somatic costs of reproduction often implicitly assume a linear trade‐off function, with somatic costs directly reflecting the fitness costs of reproduction. However, it is not clear that trade‐offs between reproduction and growth should always result in direct, linear costs to future reproductive fitness.

### 1. Costs via the male sex role

At first glance, the study of dioecious plants might appear to simplify the estimation of reproductive costs arising from the male sex role. Comparisons between growth, flowering, and survival of postreproductive females and males would appear to enable the examination of differences in reproductive costs between the sexes on the same scale. However, as noted by Midgley (2022), differences in growth or flowering are not necessarily evidence for differences in fitness between the sexes. For example, faster growth by males than females does not mean that males have greater fitness. Differences in the costs of reproduction between the sexes require demonstrating a difference in the expected future fitness of females vs males (Box 1; e.g. Ward *et al*., 2009). For comparisons of costs of reproduction between females and males, we therefore recommend a focus on traits that are more directly related to fitness. For example, comparisons between age‐specific schedules of reproductive investment and survival could point to differences in the value of current vs future reproduction between the sexes (Sherman *et al*., 2019). Moreover, we recommend against the study of somatic costs of reproduction in dioecious plants, at least if the goal of the study is to compare costs of reproduction between the sexes. Even when multiple resource types are studied and differences in somatic costs between the sexes are apparent (Harris & Pannell, 2008; Van Drunen & Dorken, 2012), whether these differences translate into differences in the lifetime reproductive fitness of females and males is not obvious.

For plants with perfect flowers, evaluating the costs of reproduction via the male sex role is also possible. In their review of the male function hypothesis (the idea that reproductive trait evolution in angiosperms is driven primarily by their fitness effects on the male sex role), Burd & Callahan (2000) outline a research programme for the analysis of investment and fitness gains via the male sex role. Because fitness gains and fitness costs are conceptually linked, the research plan outlined by Burd & Callahan (2000) would be similarly effective at identifying costs of reproduction via the male sex role. Specifically, analysis of the effects of producing fruiting and nonfruiting flowers on subsequent growth, flowering, and survival could reveal the direct and indirect effects of the male sex role on the costs of reproduction. However, the progress made in examining fitness gains via the male sex role (Stephens *et al*., 2020; Barbot *et al*., 2022; Hou *et al*., 2024) in the manner suggested by Burd & Callahan (2000) has not been matched by equally detailed analyses of the fitness costs of reproduction via the male sex role.

### 2. The shape of the trade‐off function

Reproductive allocations are made in units of resources, but reproductive costs are paid in units of fitness. Because units of nutrients or biomass are not expected to be equivalent to units of fitness, there is no general expectation for a linear association between allocations and costs (Calvo & Horvitz, 1990). The shape of these associations between allocations and costs – referred to as the trade‐off function – has rarely been examined (Reekie *et al*., 2002; Sletvold & Ågren, 2015). However, doing so could provide important insights into the evolution of plant life histories. For example, a concave‐down trade‐off function might favour plants with low reproductive allocations, particularly if those plants maintain high residual reproductive values after reaching reproductive maturity. Thus, we might expect a concave‐down trade‐off function for long‐lived plants. By contrast, plants with relatively high reproductive allocations (e.g. short‐lived perennials) might be more effectively characterized by convex cost functions (not drawn in Fig. B1), with greater expected fitness returns from investments in current vs future reproduction. Clearer connections between estimates of somatic costs and their consequences for plant fitness will fill this knowledge gap and provide insights into the mechanisms by which reproductive allocations filter through levels of biological organization.

### 3. Future considerations

The large number of studies retrieved in our systematic review underscores the important role costs of reproduction are thought to play in the ecology and evolution of plant populations. Since the previous review by Obeso (2002), there are now many more direct estimates of the effects of reproductive investments on fitness (e.g. Horvitz *et al*., 2010; Miller *et al*., 2012), and a clearer understanding that the cost function can be nonlinear (Sletvold & Ågren, 2015). At the same time, fuzzy thinking about the causes of sexual dimorphisms vs reproductive trade‐offs appears to have impeded progress in understanding the costs of reproduction in general, and for the estimation of costs paid via the male sex role in particular.

The broad range of approaches used to study costs of reproduction demonstrates that reproductive trade‐offs have wide‐ranging effects on plant growth and fitness. At the same time, this variation complicates more targeted meta‐analyses of hypotheses related to costs of reproduction in plants. For example, we generally expect long‐lived plants to have weaker costs of reproduction than plants with shorter generation times (Silvertown & Charlesworth, 2001). However, this pattern is not apparent when using simple binary scores of reproductive costs and plant longevity (Fig. 2). The standardized reporting of the magnitude of reproductive trade‐offs would enable meta‐analytical approaches for testing hypotheses related to the costs of reproduction. For tests of differences in reproductive costs between the sexes, a theoretical analysis of the conditions under which costs of reproduction can diverge between the female and male sex roles of dioecious plants could further clarify whether and when such differences should occur, and could also indicate how best to conduct empirical tests.

Progress will also be made by more clearly accounting for the effects of perenniality and clonality on current vs future reproductive fitness. On the one hand, perennial survival and clonal propagation could affect fitness indirectly via their effects on expected future (sexual) reproduction and gene transmission across generations. On the other hand, they could directly affect fitness via their effects on gene transmission through time. Survival plays a central role in regulating the trade‐off between current and future reproduction, indicating that at least when studying costs of reproduction, survival should be considered to have indirect effects on fitness via effects on future reproduction. Indeed, by evaluating trade‐offs between reproduction and survival or clonality, most of the empirical research on reproductive costs has implicitly assumed that survival and clonal propagation have indirect effects on plant fitness. By contrast, if survival and clonality have direct effects on fitness (i.e. by enabling gene transmission into the future), they are necessarily elements of reproductive investment, not reproductive costs. No studies have explicitly included survival as a component of current reproduction. However, among the subset of studies using estimates of λ to examine costs of reproduction, clonal propagation has been included in the estimation of λ and, therefore, as a component of reproduction. Because these kinds of studies hold the most promise in terms of understanding the fitness costs of reproduction, clarity on how survival and clonality contribute to current vs reproductive fitness is needed. Our view is that gene transmission across generations (not time) is most consistent with the processes shaping the evolution of plant life histories. If so, perenniality and clonality should not be considered components of (current) reproduction.

Costs of reproduction remain a central concept in plant ecology and evolution, and studies conducted over the past 20+ years have laid a solid foundation upon which a deeper understanding of the costs of reproduction can be built. Arguably, the biggest change over that time has been the increase in the number of studies using direct estimates of fitness to study reproductive costs (e.g. Horvitz *et al*., 2010). The collection of studies now available demonstrates that reproductive costs are a nearly ubiquitous feature of plants, and that costs are most clearly identified when variation in plant resource status is either experimentally controlled or otherwise accounted for when studying reproductive costs.

## Competing interests

None declared.

## Author contributions

MED led manuscript writing and data analysis. MK and MS contributed equally to manuscript writing. All authors contributed equally to planning the review, to reading the papers, and to data collection.

## Disclaimer

The New Phytologist Foundation remains neutral with regard to jurisdictional claims in maps and in any institutional affiliations.

## Supporting information


**Methods S1** Systematic review of the costs of reproduction in plants.
**Table S1** Logistic regression of associations between study design and the detection of costs.
Please note: Wiley is not responsible for the content or functionality of any Supporting Information supplied by the authors. Any queries (other than missing material) should be directed to the *New Phytologist* Central Office.
