# Supplementary material for: Costs of reproduction in flowering plants
Source: New Phytol. 2025 May 7;247(1):55–70. doi: 10.1111/nph.70166 (PMC12138173; doi:10.1111/nph.70166)
Supplement: Supplementary file 1 — Methods S1 Systematic review of the costs of reproduction in plants. Table S1 Logistic regression of associations between study design and the detection of costs. Please note: Wiley is not responsible for the content or functionality of any Supporting Information supplied by the authors. Any queries (other than missing material) should be directed to the New Phytologist Central Office. [file NPH-247-55-s001.docx]

## *New Phytologist* Supporting Information

Article title: Costs of reproduction in flowering plants

Authors: Marcel E Dorken, Mark van Kleunen, and Marc Stift

Article acceptance date: 30 March 2025

The following Supporting Information is available for this article:

**Methods S1** Systematic review of the costs of reproduction in plants

**Table S1** Logistic regression of aspects of study design and the detection of costs

**Methods S1
Systematic review of the costs of reproduction in plants**

We searched the Web of Science Core Collection on Feb. 13 2023 using the following search phrase: (ALL=("costs of reproduction" OR "cost of reproduction" OR "reproductive cost" OR "reproductive costs")) AND ALL=(plant OR tree OR shrub OR perennial OR dioec* or monoec* OR flower OR pollen OR ovule)) AND PY=(2002-2023). We added any articles that cited Obeso (2002) and that did not appear in our first search by using the Web of Science Cited Reference Search tool (also on Feb. 13 2023), and merging duplicate records.

Our initial search returned 1149 unique records. We also added records listed in Obeso’s (2002) Appendix 2 (all dioecious and other gender dimorphic plants considered in that review), yielding a total of 1219 records. From this list, we excluded conference proceedings, papers that referred to but did not measure or report reproductive trade-offs (including theoretical papers, review papers, opinion papers), and those that studied non-angiosperms. We also excluded papers that examined allometric relationships or that compared females and males without directly measuring reproductive trade-offs. To mitigate bias associated with the initial screening of articles, we used software that deployed a machine-learning algorithm to sort the records according to their fit with the inclusion criteria (van de Schoot et al., 2021). The algorithm was initially trained using a set of 10 suitable (according to our inclusion criteria) and 10 unsuitable records from the list of records obtained. The program presents articles to be screened showing only the article title and abstract in order of their fit, which is updated as articles are screened. After screening, a total of 178 studies representing 177 different species were included in our review.

We categorized studies and noted whether the data obtained addressed knowledge gaps in the understanding of reproductive costs. Papers were scored as having evaluated dynamic changes in reproductive costs if trade-offs were evaluated at multiple points in the same growing season. Papers were scored as experimental if experimental manipulation could have affected the expression of somatic and/or demographic costs of reproduction. Experimental studies were further grouped as to whether aspects of reproduction and/or growth were manipulated. For experimental studies or those based on observations of unmanipulated plants, we noted whether plants were studied in natural populations or controlled conditions (including garden experiments). Artificial selection experiments and quantitative trait locus (QTL) studies were scored as experimental because they involved (indirect) manipulations of reproductive investment (e.g., Delph et al., 2005; Ben Sadok et al., 2013). By contrast, studies of patterns of gene-expression before and after reproduction (e.g., Cossard et al., 2019) were scored as observational.

We categorized studies as having tested the ecological costs of reproduction if they examined fitness reproductive trade-offs arising from interactions with other organisms (e.g., increased herbivory or competition for flowering versus non-flowering plants). We also noted whether studies involved the use of long-term monitoring, which we define here as the study of plants in at least three consecutive years. Finally, unless authors provided an alternative explanation for how size-dependent costs of reproduction were tested, a study was classed as having tested for size-dependency of reproductive costs if a size by reproductive status interaction term was included in their statistical model(s) (Shelton, 2010).

We scored studies as having assessed somatic or demographic costs of reproduction by considering the types of measurements made and the time-span of the study. We first noted whether the reproductive trade-offs studied involved measurements made within a single growing season or between seasons. Studies of the demographic costs of reproduction involve trade-offs between current and future reproduction and/or survival, and typically therefore, the measurement of traits that span at least two seasons. A small number of studies using alternative approaches were also scored as having assessed fitness-based trade-offs (Thompson & Eckert, 2004; Oddou-Muratorio et al., 2021). For studies examining within-season costs, we noted whether trade-offs between reproduction and growth (including physiological performance) or biomass and nutrient allocations were examined. Some studies of between-season costs also examined the trade-off between current reproduction and plant size in the subsequent year. Studies of reproduction-growth (or allocation) trade-offs were categorized as studies of somatic costs, while studies of trade-offs between current and future reproduction and/or survival were scored as studies of demographic costs.

To enable categorization of species-level features, we tabulated all species included among the 178 studies. Based on information provided in the study, or found by searching other studies on the same species, we noted species that are dimorphic for gender (i.e., populations with at least one unisexual phenotype, male or female, including dioecy, gynodioecy and androdioecy). Finally, we examined the representation of different Angiosperm families and how this varies with the species richness of those families by retrieving data from (Ferrer et al., 2023), who tabulated species richness data from the Angiosperm Phylogeny Website (Stevens, 2017). Unlike Obeso (2002), we included domesticated plants in our review. Even though domesticated plants have typically been selected for higher yields, studies of domesticated plants include those that specifically evaluated varieties with contrasting patterns of growth versus reproductive investment (e.g., Rosati et al., 2018) and might therefore provide insights into how variation in reproductive allocations affect the costs of reproduction. Data manipulation and visualization was conducted using R [v. 4.4.2; R Core Team (2024); data and R scripts are available at <https://doi.org/10.6084/m9.figshare.26388499>].

**References**

**Ben Sadok I, Celton J, Essalouh L, El Aabidine A, Garcia G, Martinez S, Grati-Kamoun N, Rebai A, Costes E, Khadari B**. **2013**. [QTL mapping of flowering and fruiting traits in olive](https://doi.org/10.1371/journal.pone.0062831). *PLOS ONE* **8**.

**Cossard GG, Toups MA, Pannell JR**. **2019**. [Sexual dimorphism and rapid turnover in gene expression in pre-reproductive seedlings of a dioecious herb](https://doi.org/10.1093/aob/mcy183). *Annals of Botany* **123**: 1119–1131.

**Delph L, Gehring J, Arntz A, Levri M, Frey F**. **2005**. [Genetic correlations with floral display lead to sexual dimorphism in the cost of reproduction](https://doi.org/10.1086/444597). *American Naturalist* **166**: S31–S41.

**Ferrer MM, Vásquez-Cruz M, Hernández-Hernández T, Good SV**. **2023**. [Geographical and life-history traits associated with low and high species richness across angiosperm families](https://doi.org/10.3389/fpls.2023.1276727). *Frontiers in Plant Science* **14**.

**Obeso JR**. **2002**. [The costs of reproduction in plants](https://livelancsac.sharepoint.com/sites/Dropbox/Documents/Notes/Projects/CostsOfReproduction/Costs%20of%20reproduction%20in%20plants/%3CGo%20to%20ISI%3E:/000177545000002). *New Phytologist* **155**: 321–348.

**Oddou-Muratorio S, Petit-Cailleux C, Journé V, Lingrand M, Magdalou J-A, Hurson C, Garrigue J, Davi H, Magnanou E**. **2021**. [Crown defoliation decreases reproduction and wood growth in a marginal European beech population](https://doi.org/10.1093/aob/mcab054). **Annals of Botany 128**: 193–204.

**R Core Team. 2024**. [*R: A language and environment for statistical computing*](https://www.R-project.org/). Vienna, Austria: R Foundation for Statistical Computing.

**Rosati A, Paoletti A, Al Hariri R, Morelli A, Famiani F**. **2018**. [Resource investments in reproductive growth proportionately limit investments in whole-tree vegetative growth in young olive trees with varying crop loads](https://doi.org/10.1093/treephys/tpy011). *Tree Physiology* **38**: 1267–1277.

**Shelton AO**. **2010**. [The origin of female‐biased sex ratios in intertidal seagrasses (Phyllospadix spp.)](https://doi.org/10.1890/09-0685.1). *Ecology* **91**: 1380–1390.

**Stevens PF**. **2017**. [Angiosperm Phylogeny Website](http://www.mobot.org/MOBOT/research/APweb/). URL: http://www.mobot.org/MOBOT/research/APweb/ Accessed June 17 2024.

**Thompson FL, Eckert CG**. **2004**. [Trade-offs between sexual and clonal reproduction in an aquatic plant: experimental manipulations vs. phenotypic correlations](https://doi.org/10.1111/j.1420-9101.2004.00701.x). *Journal of Evolutionary Biology* **17**: 581–592.

**van de Schoot R, de Bruin J, Schram R, Zahedi P, de Boer J, Weijdema F, Kramer B, Huijts M, Hoogerwerf M, Ferdinands G, et al. 2021**. [An open source machine learning framework for efficient and transparent systematic reviews](https://doi.org/10.1038/s42256-020-00287-7). **Nature Machine Intelligence** 3: 125–133.

**Notes S1** **Logistic regression**

Table S1. Logistic regression of the association between various aspects of study design and the detectability of costs of reproduction. Values are the parameter estimates (SE) associated with each of the different aspects of study design included in the model.

|  | |
| --- | --- |
|  | Dependent variable: |
|  |  |
|  | Reproductive costs |
|  | |
| Observational studies | 0.187 |
|  | (0.303) |
|  |  |
| Natural populations | -1.545*** |
|  | (0.382) |
|  |  |
| Woody plants | 0.452 |
|  | (0.292) |
|  |  |
| Within-season costs | 0.249 |
|  | (0.269) |
|  |  |
| Whole-plant studies | -0.865** |
|  | (0.337) |
|  |  |
| Constant | 1.548*** |
|  | (0.527) |
|  |  |
|  | |
| Observations | 280† |
| Log Likelihood | -178.429 |
| Akaike Inf. Crit. | 368.859 |
|  | |
| *p<0.1; **p<0.05; ***p<0.01 | |

†The vast majority of studies reported results that were consistent with the expression of costs of reproduction, causing the logistic regression to be strongly unbalanced. To calculate the parameter estimates reported in the table, we used a re-sampling procedure to create a rebalanced dataset with upsampled instances of studies reporting no costs of reproduction. For analyses of both the original and upsampled datasets, the model intercept and whether studies were conducted in natural populations were statistically significant. Only when analysing resampled data were studies of entire plants found to be associated with the detection of costs of reproduction. Additional details, including the code used to conduct both logistic regressions with parameter estimates for the analysis of the original (non upsampled) data are provided at <https://doi.org/10.6084/m9.figshare.26388499>.
